# mRNA, lncRNA, and circRNA expression profiles in a new aortic dissection murine model induced by hypoxia and Ang II

**DOI:** 10.3389/fcvm.2022.984087

**Published:** 2022-10-26

**Authors:** Yuanyuan Li, Xiaozhu Ma, Shuai Mei, Yueping Ji, Dong Wang, Liqun He, Dating Sun, Jiangtao Yan

**Affiliations:** ^1^Department of Cardiology, Tongji Hospital, Tongji Medical College, Huazhong University of Science and Technology, Wuhan, China; ^2^Department of Rheumatology and Immunology, Tongji Hospital, Tongji Medical College, Huazhong University of Science and Technology, Wuhan, China; ^3^Department of Cardiology, Wuhan No. 1 Hospital, Wuhan Hospital of Traditional Chinese and Western Medicine, Wuhan, China

**Keywords:** aortic dissection, obstructive sleep apnea, hypoxia, bioinformatics, aortic structure, apoptosis, inflammation

## Abstract

**Background and aims:**

Aortic dissection (AD) is a cardiovascular emergency with degeneration of the aortic media. Mounting evidence indicates obstructive sleep apnea (OSA) as an independent risk factor for AD development with unknown mechanisms. This study aims to establish a stable murine model of OSA-related AD (OSA-AD) and uncover the potential changes in gene transcripts in OSA-AD.

**Materials and methods:**

ApoE^–/–^ mice were exposed to the chronic intermittent hypoxia (CIH) system combined with Ang II administration to establish the OSA-AD model. Pathological staining was performed to exhibit the physiological structure of the mouse aorta. The SBC mouse ceRNA microarray was used to identify significantly differentially expressed (DE) mRNAs, DE long-non-coding RNAs (DElncRNAs), and DE circular RNAs (DEcircRNAs) in OSA-AD tissues. Subsequently, bioinformatics analysis, including Gene Ontology (GO), Kyoto Encyclopedia of Genes and Genome (KEGG), and protein–protein interaction (PPI) analyses, were performed to evaluate the function of the significantly differentially expressed transcripts (DETs). The hub genes were confirmed using quantitative real-time polymerase chain reaction (qRT-PCR).

**Results:**

ApoE^–/–^ mice exposed to CIH and Ang II showed a high ratio of aortic accident (73.33%) and significant aortic diameter dilatation (1.96 ± 0.175 mm). A total of 1,742 mRNAs, 2,625 lncRNAs, and 537 circRNAs were identified as DETs (LogFC ≥ 1.5 or ≤ –1.5, *P* < 0.05). GO and KEGG analyses demonstrated that the differentially expressed mRNAs (DEmRNAs) were most enriched in cell proliferation, migration, apoptosis, inflammation, and hypoxia-related terms, which are closely related to aortic structural homeostasis. The PPI network contained 609 nodes and 934 connections, the hub genes were highlighted with the CytoHubba plugin and confirmed by qRT-PCR in AD tissues. KEGG pathway analysis revealed that the *cis*-regulated genes of DElncRNAs and circRNAs-host genes were enriched in aortic structural homeostasis-related pathways.

**Conclusion:**

Our findings help establish a *de novo* OSA-AD animal model using ApoE^–/–^ mice. Many DEmRNAs, DElncRNAs, and DEcircRNAs were screened for the first time in OSA-AD tissues. Our findings provide useful bioinformatics data for understanding the molecular mechanism of OSA-AD and developing potential therapeutic strategies for OSA-AD.

## Introduction

Aortic dissection (AD) is a highly fatal cardiovascular emergency that is characterized by the formation of an intimal flap within the aortic wall, causing blood to flow between the aortic layers and creating a false lumen with a narrow true lumen (1). Epidemiological studies have shown that the true incidence of AD may range from 2.6 to 3.5 cases per 100,000 annually in 65–75-year-old individuals (2, 3). Histopathological studies have revealed that cystic medial necrosis, elastic fiber loss, and interconnecting elastic fiber loss are the main changes associated with AD (2). Etiological studies have shown that obstructive sleep apnea (OSA), genetic mutations, hypertension, true aortic aneurysms, inflammatory aortic diseases, pregnancy and steroid use, and atherosclerosis are important causes of AD (2).

Obstructive sleep apnea is considered an independent risk factor for cardiovascular disease (4, 5), which is closely related to hypertension, pulmonary hypertension, stroke, heart failure, atrial fibrillation, and coronary heart disease (4, 6). Additionally, OSA is relevant to increased all-cause mortality and cardiovascular mortality (4). In the last two decades, epidemiological studies have confirmed that OSA is an independent risk factor for AD, indicated by the higher OSA index in patients with AD (7–10). Notably, the aortic diameter was substantially larger in patients with OSA than healthy individuals (11, 12). Nevertheless, the molecular mechanism of OSA-AD remains largely unknown. According to a hypothesis, the negative intrathoracic pressure in OSA may increase the transmural pressure of the aortic wall, resulting in vascular damage. An increase in blood pressure may be necessary to initiate the presence of an entry tear (13). A recent study reported that OSA facilitates the production of reactive oxygen species (ROS), which activate HIF-α to promote the expression of matrix metalloproteases that destroy the aortic structure (14). Recent studies demonstrated that long-non-coding RNAs (lncRNAs) and circular RNAs (circRNAs) play crucial roles in diverse cellular and physiological functions (15, 16). Although a few lncRNAs and circRNAs have been implicated in AD (17–19), the expression and function of a large number of differentially expressed (DE) lncRNAs and DEcircRNAs in AD have not been explained. Additionally, the expression and function of lncRNAs and circRNAs in OSA-AD have not been reported. Given the difficulty of understanding how OSA affects the development of AD, there are currently no effective therapeutic strategies for OSA-AD intervention or prevention. Therefore, evaluating the molecular mechanism is urgently needed for establishing therapeutic strategies against OSA-AD.

In this study, we first established a stable OSA-AD mouse model. We induced OSA-AD in ApoE^–/–^ mice using the chronic intermittent hypoxia (CIH) system and Ang II administration. Subsequently, the SBC mouse ceRNA microarray was used to investigate significant DEmRNAs, DElncRNAs, and DEcircRNAs in OSA-AD tissues. Then, we performed bioinformatics analyses to identify the potential roles of DEmRNAs, DElncRNAs, and DEcircRNAs in OSA-AD.

## Materials and methods

### Human samples

Aortic tissues were obtained from patients with thoracic aortic dissection (AD samples) and patients undergoing heart transplantation (normal controls). All samples were stored in liquid nitrogen or paraformaldehyde. All protocols using human specimens were approved by the Human Research Ethics Committees of Tongji Hospital, Tongji Medical College, Huazhong University of Science and Technology, and informed consent was obtained from patients or their family members.

### Animals

All animal experiments were conducted according to the Guide for the Care and Use of Laboratory Animals published by the US National Institutes of Health (NIH Publication No. 85–23, revised 1996) and the Public Health Service Policy on Humane Care and Use of Laboratory Animals. Animals were anesthetized with 1% pentobarbital before experiments were conducted. Five mice unexpectedly died before the exposure endpoint. Mice were raised in temperature-controlled cages in a 12-h light–dark cycle with a normal diet. This study was approved by the animal care facility of Tongji Medical College, Wuhan, China. ApoE^–/–^ (C57BL/6 background) mice were provided by the Charles River Laboratories (MA, USA) and were housed at the animal care facility of Tongji Medical College under specific pathogen-free conditions. All animal work was approved by the Institutional Animal Research Committee of Tongji Medical College.

### Mice obstructive sleep apnea model

The OSA mice model was established as follows. In brief, ApoE^–/–^ mice were treated in the CIH system; room air (RA) condition was used as a control. The flow of oxygen and nitrogen was regulated by the gas control system into the chamber at a specified time and concentration. During an intermittent hypoxia cycle, nitrogen was transferred to the chamber to achieve 6% O2 for 60 s. Then, oxygen was delivered into the chamber to achieve 21% O_2_ concentration for 60 s. Mice were housed in the chamber for 30 cycles/h, 8 h/day for 7 day/week and 4 weeks. In the control group, mice were placed in a chamber with 21% O_2_ for the same time cycle. All mice were euthanized at the end of the experiment.

### Establishment of the aortic dissection model

Eight-week-old ApoE^–/–^ mice were implanted with a microosmotic pump (Model 2004, Durect Corporation, Cupertino, CA, USA), which was filled with 2.5 μg/kg/min Ang II (Sigma-Aldrich). Saline was used as a control for Ang II. The mice were given the treatment for 14 days. Forty-eight 8-week-old ApoE^–/–^ mice were randomly grouped as follows: saline infusion with RA (*n* = 6); Ang II infusion with RA (*n* = 17); Ang II infusion plus CIH (n = 16); and saline infusion plus CIH (*n* = 9). Two weeks after CIH, mice were infused with Ang II for another 2 weeks in the CIH groups.

### Aortic dissection and aortic rupture evaluation in mice

Animals were anesthetized at the end of all interventions. For AD quantification, we evaluated the maximum width of the aorta (ascending arch, descending thoracic, and abdominal aortic segments) with the Image Pro Plus software (Media Cybernetics, Bethesda, MD, USA). AD in mice was defined as the presence of hematoma or the presence of layer separation within the aortic wall detected on gross or histological appearance.

### Immunohistochemical staining

Aortas were fixed in 4% paraformaldehyde and embedded with paraffin for histological analysis. Then the aortas were sectioned into 3-μm cross-sections and stained with hematoxylin and eosin and Verhoeff-Van Gieson (EVG). Negative controls were used to validate the specificity of each immunostaining.

### Immunofluorescence staining

After incubating with 10% FBS, the sections were incubated with primary antibodies overnight at 4°C, then, the primary antibodies were replaced by secondary antibody labels and cell nuclei were stained with DAPI. Primary antibodies: α-SMA (cat. no. Ab5694; dilution: 1:50; Abcam), CASP3 (cat. no. ab32351; dilution: 1:50; Abcam), IL-1β (cat. no. ab254360; dilution: 1:50; Abcam). Secondary antibodies: Alexa Fluor^®^ 568-conjugated goat anti-rabbit immunoglobulin G (cat. no. A-11034; dilution, 1:250; Thermo Fisher Scientific), and Alexa Fluor^®^ 488-conjugated goat anti-mouse immunoglobulin G (cat. no. A32727; dilution, 1:250; Thermo Fisher Scientific). Images were obtained by E2000U microscope (Nikon Corporation, Tokyo, Japan).

### RNA extraction, SBC-ceRNA microarray, and data preprocessing of differentially expressed transcripts

Total RNA from mouse aortic tissues, including three normal tissues (induced by RA + saline) and 3 AD tissues (induced by CIH + Ang II), was extracted using TRIzol reagent (Invitrogen, CA, USA) according to the manufacturer’s protocol. The NanoDrop ND-2000 (Thermo Scientific, MA, USA) was used to quantify total RNA, and the Agilent Bioanalyzer 2100 (Agilent Technologies) was used to evaluate the integrity of the total RNA. The total RNA was hybridized to SBC mouse (4 × 180 K) ceRNA microarray by SHBIO^1^ to identify significant differentially expressed transcripts (DETs), including mRNAs, lncRNAs, and circRNAs. Sample labeling, microarray hybridization, and washing were performed following the standard protocols of the manufacturer. The extraction software (version10.7.1.1, Agilent Technologies) was used to analyze array images to get raw data. GeneSpring was used to complete the basic analysis of the raw data. Significant DETs between normal and AD tissues were identified and retained by screening for a log (fold change) ≥ 1.5 or ≤ –1.5 and *P* < 0.05. To visually display the DETs, volcano and heatmap analysis was performed for significant DETs using imageGP.^2^

### Gene ontology and kyoto encyclopedia of genes and genome pathway enrichment analysis

Gene ontology (GO) analysis was performed using DEmRNAs, which involve cell composition (CC), biological process (BP), and molecular function (MF). The kyoto encyclopedia of genes and genomes (KEGGs) pathway analysis offers biological pathways of DEmRNA genes in AD tissues induced by CIH and Ang II. The GO and KEEG analysis and enrichment plots were constructed using the online biological function database bioinformatics^3^ and DAVID.^4^ The enrichment index with *P* < 0.05 was considered statistically significant.

### Protein–protein interaction network construction and hub gene identification

To explore possible protein–protein interaction (PPI) network interactions, the DEmRNAs were mapped to the Search Tool for the Retrieval of Interacting Genes/Proteins database (STRING^5^). PPI pairs were extracted with a high required interaction score > 0.9. The interactions were downloaded from STRING and used to construct a PPI network by Cytoscape software platform (3.9.0). Nodes with a higher degree of connectivity were considered important in maintaining the stability of the PPI network; therefore, the CytoHubba plugin for Cytoscape software was used to identify the top 20 hub genes.

### Quantitative reverse transcription-polymerase chain reaction

As described in Sun et al. (20), total RNA was extracted from aortic tissues of mice and human. Then the RNA was converted to cDNA using the HiScript II Q RT SuperMix for qPCR (+gDNA wiper) (R223-01, Vazyme, China). qPCR was performed using the ChamQ SYBR qPCR Master Mix (#Q311-02, Vazyme, China). *Gapdh/GAPDH* was used as an endogenous control. Data analyses were performed using the SDS 2.2.2 software. The primers are listed in Supplementary Tables 1, 2.

### *Cis*-regulatory network construction of differentially expressed long-non-coding RNAs

Long-non-coding RNAs play a role in modulating adjacent gene expression to produce biological effects *via* a *cis*-regulatory network. Therefore, the prediction of potential target genes of DElncRNAs was performed. DElncRNAs *cis*-regulated target genes were mainly predicted based on location distribution, with the lncRNA *cis*-regulated network defined as constructed by DElncRNAs and DEmRNAs within 10 kbp of the chromosome. Pearson’s correlation analysis was performed on *cis*-lncRNA–mRNA pairs (*P* < 0.05). The *cis*-lncRNA regulatory network was visualized using the Cytoscape software platform (3.9.0).

### Statistical analysis

All data were presented as mean ± standard deviation (SD) of three independent experiments with the software GraphPad Prism 7. Significance was assessed by one-way ANOVA followed by Tukey’s test. All statistical evaluations were performed using SPSS 22.0 (SPSS Inc., Chicago, IL, USA). A *P*-value < 0.05 was considered statistically significant.

## Results

### The physiological structure of the aorta deteriorated in obstructive sleep apnea-related aortic dissection mice

**FIGURE 1:**
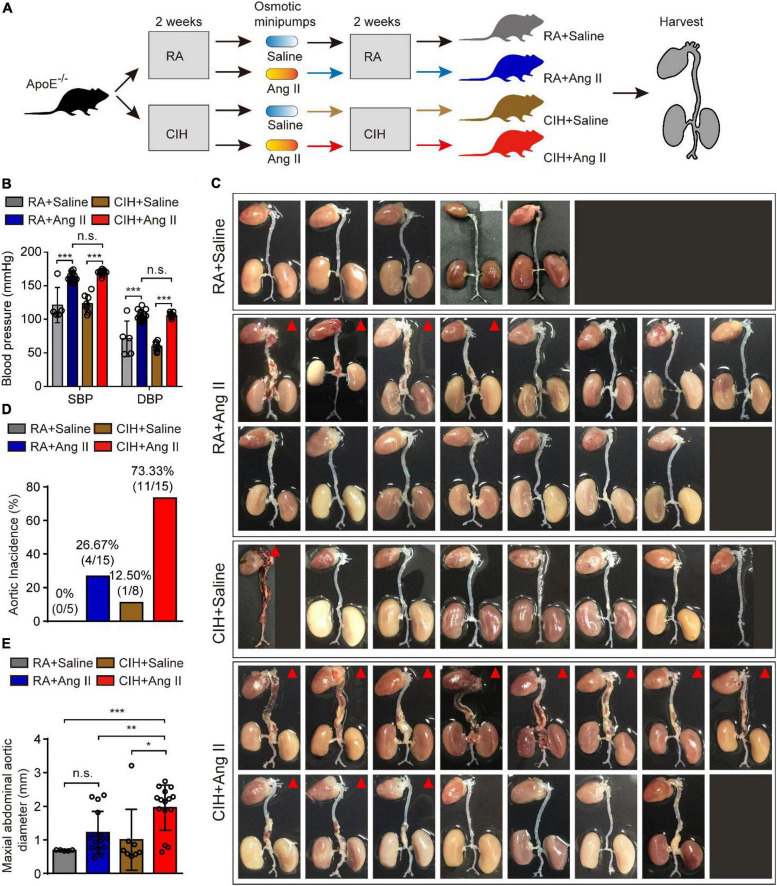
The establishment of the aortic dissection (AD) mouse model induced by chronic intermittent hypoxia (CIH) and Ang II. **(A)** Diagram illustrates the experimental design for establishing the AD model. Briefly, ApoE^– /–^ mice were preprocessed in the CIH system [room air (RA)-pretreated mice were used as control] for 2 weeks. Next, mice were implanted with microosmotic pumps filled with Ang II (saline was used as control). Then, mice received the CIH treatment (RA for control mice) for 2 weeks. Finally, aortic tissues were obtained from all animals. **(B)** The blood pressure of mice after CIH/RA and Ang II/saline administration. **(C)** Whole aorta tissue from the indicated mice after CIH/RA and Ang II/saline administration. The red triangle shows mice with aortic accident. **(D)** The histogram shows the ratio of aortic accident in the four groups of mice. **(E)** The histogram shows the maximal abdominal aortic diameter in the four groups of mice. For panels **(B–E)**, *n* = 5–15 mice per group; n.s., not significant; *P < 0.05, **P < 0.01, ***P < 0.001.

**FIGURE 2:**
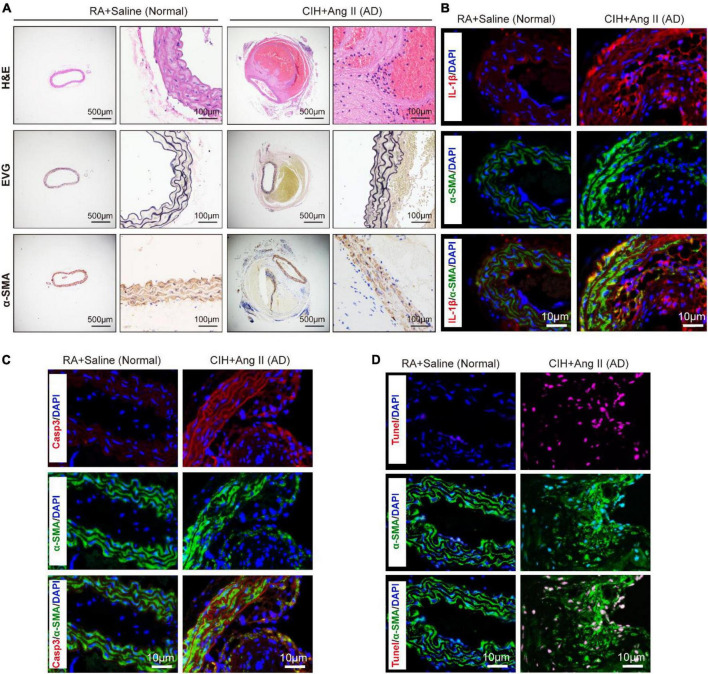
Transverse section of pathological aortic tissues. **(A)** Representative images exhibit aortic sections stained with H&E (first row) and EVG (second row). The third row shows aortic sections stained with the α-SMA antibody. **(B–D)** Representative images of immunofluorescence staining demonstrate Il-1β **(B)**, Casp3 **(C)** expression and Tunel positive cells **(D)** in aortic tissues of chronic intermittent hypoxia (CIH) and Ang II induced AD and control mice. Scale bar: for panel **(A)**, left 500 μm and right 100 μm; for panels **(B–D)**, 10 μm. EVG, Verhoeff-Van Gieson.

To evaluate the role of OSA in the development of AD, we simulated OSA using the CIH system and designed a scheme for establishing the OSA-AD animal model (Figure 1A). Briefly, after 2 weeks of preprocessing under CIH, ApoE^–/–^ mice were implanted with microosmotic pumps filled with Ang II subcutaneously. Then, the mice received CIH treatment for another 2 weeks. At the end of the experiment, mice blood pressure was measured, as expected, the blood pressure was increased in mice treated with Ang II, but no affected by hypoxia (Figure 1B). Besides, the mice heart rate in four groups showed no significance (Supplementary Figure 1A). Mice aortas were obtained from the mice (Figure 1C), and the rate of aortic accidents and aortic diameters were calculated in the four groups. Mice induced by RA and saline showed no aortic accidents (0%, 0/5); however, aortic accident rate in mice induced with RA and Ang II (26.67%, 4/15) showed an increase. Importantly, the aortic accident rates in mice induced by CIH combined with saline or Ang II were 12.5% (1/8) and 73.33% (11/15), respectively (Figure 1D). The maximal diameters of the aorta were 0.69 ± 0.006 mm (RA + saline), 1.22 ± 0.163 mm (RA + Ang II), 1.00 ± 0.320 mm (CIH + saline), and 1.96 ± 0.175 mm (CIH + Ang II) (Figure 1E). In addition, we have conducted β-Aminopropionitrile (BAPN)-induced AD in C57 mice (Supplementary Figure 1B), the incidence of AD is too low in this model and we failed to do further analysis. Next, histological analysis (using H&E and EVG staining) of the aorta of mice induced with CIH and Ang II showed that the aortic pseudocoel was inflated and accompanied a thinner aortic wall (Figure 2A). Immunohistochemical staining with the anti-α-SMA antibody showed collagen loss in AD tissues, which was induced by CIH and Ang II (Figure 2A). Importantly, the immunofluorescence staining with Casp3, Tunel and Il-1β were showed the cell apoptosis and inflammatory response were triggered in aortic tissues induced by CIH/Ang II (Figures 2B–D). Taken together, the aortic structure of the mice was destroyed and cell apoptosis and inflammatory response were triggered in mouse aortic tissues induced by CIH/Ang II.

### Identification of differentially expressed transcripts in aortic dissection tissues induced by chronic intermittent hypoxia and Ang II

**FIGURE 3:**
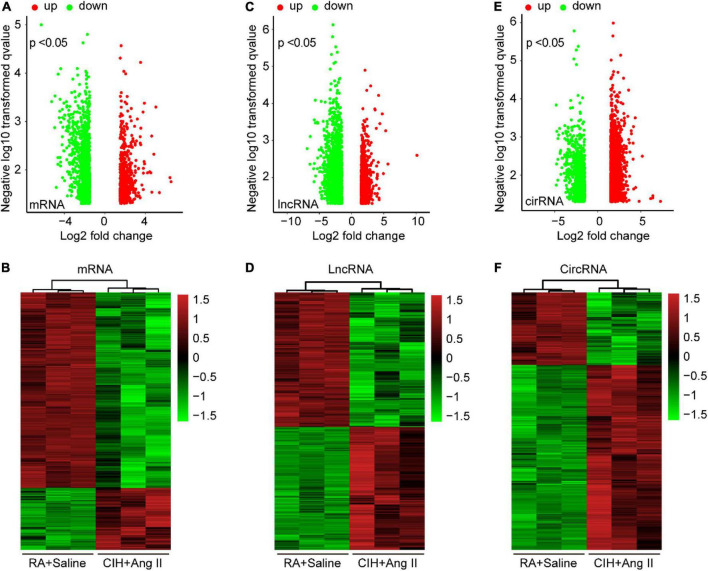
Volcano plots and heatmaps of differentially expressed (DE) mRNAs, long-non-coding RNAs (lncRNAs), and circular RNAs (circRNAs) in aortic dissection (AD) and normal tissues. **(A,C,E)** Volcano plots of DE mRNAs **(A)**, lncRNAs **(C)**, and circRNAs **(E)** in the ceRNA microarray; green and red points represent down- and upregulated transcripts, respectively; **(B,D,F)** The heatmap of DE mRNAs **(B)**, lncRNAs **(D)**, and circRNAs **(F)** in the ceRNA microarray. From panels **(A–F)**, log (fold change) ≥ 1.5 or ≤ -1.5, *P* < 0.05.

To identify potential change in gene transcripts, including mRNAs, lncRNAs, and circRNAs in AD tissues induced by CIH and Ang II, we performed the SBC-ceRNA microarray assay. Transcripts with log (fold change) ≥ 1.5 or ≤ -1.5 and *P* < 0.05 were identified as being DE. According to the volcano plots and heatmaps (Figure 3), 1742 mRNAs (Figures 3A,B), 2625 lncRNAs (Figures 3C,D), and 537 circRNAs (Figures 3E,F) were identified as being DE, including 1152 downregulated mRNAs, 590 upregulated mRNAs, 1,507 downregulated lncRNAs, 1,118 upregulated lncRNAs, 167 downregulated circRNAs, and 370 upregulated circRNAs. The top 10 upregulated and downregulated genes of DEmRNAs, DElncRNAs, and DEcircRNAs are listed in Tables 1–3, respectively.

**TABLE 1:** The top 10 upregulated and downregulated genes of DEmRNAs.

Gene symbol	LogFC	*p*-values	Gene symbol	LogFC	*P*-values
Saa3	6.537	0.018	Perp	–4.425	0.000
Spp1	6.433	0.015	Npnt	–4.455	0.001
Lars2	5.387	0.030	Cytl1	–4.500	0.001
Clec4d	5.033	0.001	Kcnab1	–4.613	0.000
Nxpe5	4.840	0.005	2210407C18Rik	–4.627	0.006
P4ha3	4.634	0.029	Dsp	–4.628	0.008
Ptx3	4.622	0.002	Mylk4	–4.795	0.023
Kap	4.534	0.012	Synpo2	–4.915	0.000
Arg1	4.429	0.011	Ppef1	–4.958	0.001
Trem2	4.205	0.012	Slc22a1	–5.255	0.000

DEmRNAs, differentially expressed mRNAs.

**TABLE 2:** The top 10 upregulated and downregulated genes of DElncRNAs.

Accession	LogFC	*p*–values	Accession	LogFC	*P*-values
NON-MMUT007296	4.195	0.000	NON-MMUT016917	–6.889	0.002
NON-MMUT109857	4.266	0.020	NON-MMUT132917	–6.449	0.004
NON-MMUT133367	4.275	0.036	ENSMUST00000181120	–6.392	0.001
ENSMUST00000143272	4.281	0.040	NON-MMUT017361	–5.870	0.006
NON-MMUT096745	4.397	0.000	NON-MMUT111310	–5.800	0.013
NON-MMUT093632	4.759	0.000	NON-MMUT111296	–5.776	0.000
NON-MMUT093631	4.939	0.008	NON-MMUT010241	–5.771	0.004
NON-MMUT094891	5.290	0.001	NON-MMUT116079	–5.620	0.000
NON-MMUT135123	5.642	0.004	ENSMUST00000131898	–5.604	0.000
NON-MMUT110746	10.148	0.003	ENSMUST00000148089	–5.581	0.009

DElncRNAs, differentially expressed lncRNAs.

**TABLE 3:** The top 10 upregulated and downregulated genes of DEcircRNAs.

CircRNA_ID	LogFC	*p*-values	CircRNA_ID	LogFC	*P*-values
mmu_circ_0008002	2.867	0.011	mmu_circ_0004366	–4.643	0.014
mmu_circ_0008306	2.931	0.003	mmu_circ_0000570	–3.815	0.001
mmu_circ_0002842	2.933	0.037	mmu_circ_0001881	–3.814	0.003
mmu_circ_0000648	2.950	0.002	mmu_circ_0016354	–3.697	0.001
mmu_circ_0000746	3.127	0.044	mmu_circ_0014597	–3.686	0.012
mmu_circ_0013434	3.242	0.011	mmu_circ_0004377	–3.600	0.008
mmu_circ_0007410	3.274	0.040	mmu_circ_0004358	–3.573	0.007
mmu_circ_0005483	3.304	0.002	mmu_circ_0009280	–3.540	0.005
mmu_circ_0001921	3.618	0.046	mmu_circ_0015838	–3.420	0.007
mmu_circ_0003720	4.404	0.021	mmu_circ_0007117	–3.370	0.012

DEcircRNAs, differentially expressed cirRNAs.

### Functional enrichment analyses of differentially expressed mRNAs

**FIGURE 4:**
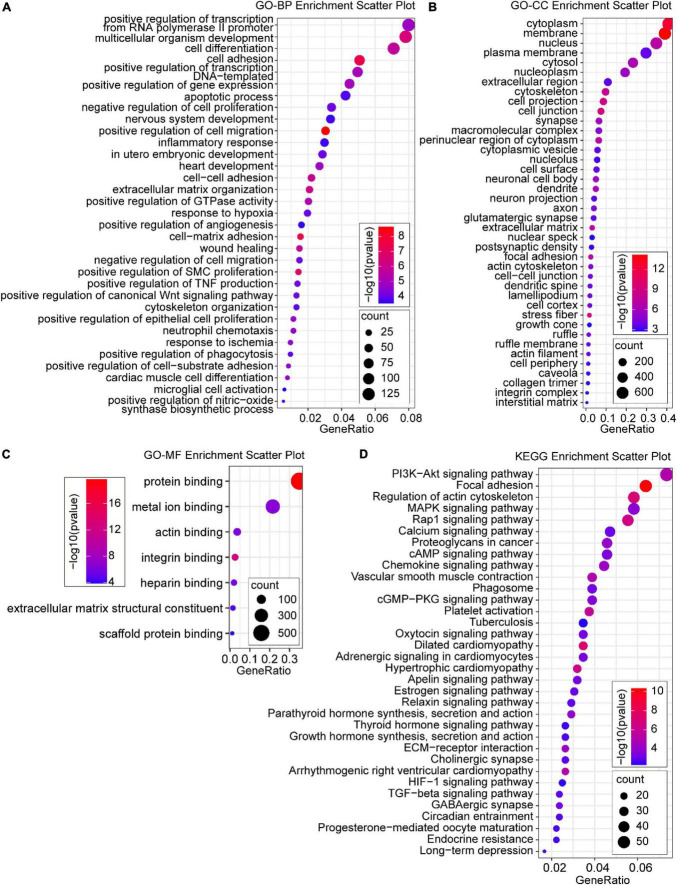
Gene ontology (GO) and Kyoto Encyclopedia of Genes and Genome (KEGG) enrichment analysis of DE mRNAs. **(A)** Significant GO biological process (BP) terms; **(B)** significant GO cellular component (CC) terms; **(C)** most significant GO molecular function (MF) terms; **(D)** enriched KEGG pathway terms. *P* < 0.05 for all significant GO terms and KEGG pathway terms.

To further reveal the functions of the DEmRNAs in the development of AD induced by CIH and Ang II, GO and KEGG analyses were performed to classify the functions of the DEmRNAs (Figure 4). GO enrichment analysis showed that 81 GO terms were enriched (FDR < 0.05) in DEmRNAs, which included 34 BP, 40 cellular components (CC), and seven molecular function (MF) ontologies (Figures 4A–C). For BP ontology, the DEmRNAs were mostly enriched in cell apoptosis, cell proliferation, cell migration, inflammation, and hypoxia-related terms (Figure 4A), including “-positive regulation of cell migration,” “negative regulation of cell proliferation,” “positive regulation of smooth muscle cell proliferation,” “response to hypoxia,” “response to ischemia,” “apoptotic process,” “inflammatory response,” “neutrophil chemotaxis,” “positive regulation of TNF production,” “positive regulation of angiogenesis,” and “-positive regulation of nitric-oxide synthase biosynthetic process.” In CC ontology, the “nucleus,” “membrane,” “cytoplasm,” “stress fiber,” “cell junction,” “extracellular matrix,” and “actin cytoskeleton” were enriched (Figure 4B). For MF ontology, the DEmRNAs were mostly enriched in “protein binding,” “integrin binding,” “metal ion binding,” “actin binding,” “heparin binding,” “extracellular matrix structural constituent,” and “scaffold protein binding” (Figure 4C). For KEGG pathway enrichment analysis, 34 GO terms were enriched (FDR < 0.05), and the DEmRNAs were most significantly enriched in “PI3K-Akt signaling pathway,” “Focal adhesion,” “Regulation of actin cytoskeleton,” “MAPK signaling pathway,” “Rap1 signaling pathway,” “Calcium signaling pathway,” “cAMP signaling pathway,” “Chemokine signaling pathway,” “cGMP-PKG signaling pathway,” “Platelet activation,” “HIF-1 signaling pathway,” and “TGF-beta signaling pathway” (Figure 4D). Importantly, these pathways were mostly related to inflammation, apoptosis, vascular activity, and hypoxia.

### Protein–protein interaction network construction and hub gene identification

**FIGURE 5:**
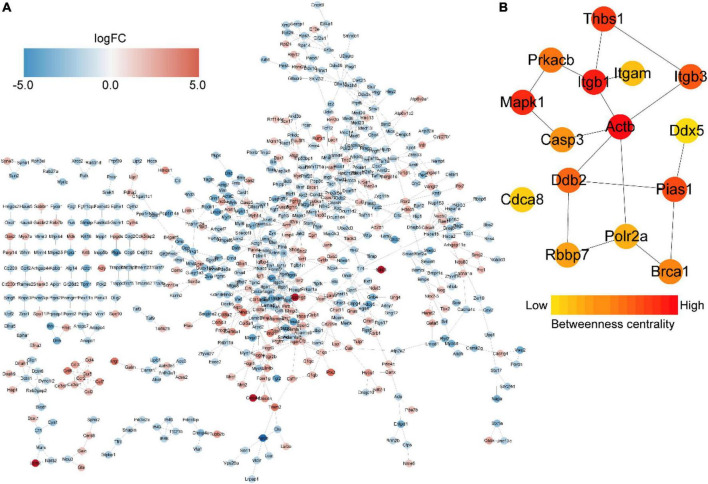
Protein–protein interaction (PPI) networks and hub genes of differentially expressed mRNAs (DEmRNAs) in aortic dissection (AD) tissues compared with controls. **(A)** PPI networks show the interaction of DEGs, with interaction scores > 0.9. The red to blue color gradient indicates an up- to downregulation of DEmRNAs, indicated by logFC. **(B)** The top 15 hub genes from DEmRNAs. The red to yellow color gradient indicates high to low centrality.

**FIGURE 6:**
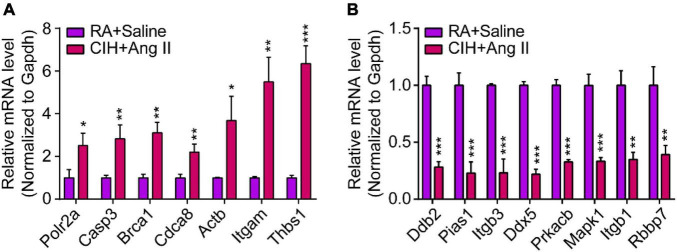
Quantitative real-time polymerase chain reaction (qRT-PCR) analysis of gene expression levels of hub genes in aortic dissection (AD) tissues induced by chronic intermittent hypoxia (CIH) and Ang II. **(A,B)** qRT-PCR analysis show relative gene expression of hub genes, including **(A)** upregulated hub genes and **(B)** downregulated hub genes. *n* = 3 per group, *P < 0.05, **P < 0.01, ***P < 0.001.

We then constructed a PPI network and evaluated the interactions through the STRING database to explore the association between the DEmRNAs. After calculation, the interaction score was set at 0.9, 609 nodes and 934 connections were generated, and the PPI network was visualized using the Cytoscape software (Figure 5A). Subsequently, the top 15 DEmRNAs with a high degree of connectivity were selected as hub genes in AD tissues induced by CIH and Ang II (Figure 5B and Table 4). We confirmed the screening results by quantitative reverse transcription-polymerase chain reaction (qRT-PCR), and the expression of these hub genes (seven upregulated and eight downregulated genes) was consistent with the ceRNA microarray (Figures 6A,B). Moreover, most upregulated hub genes were related to inflammation and apoptosis, including *Casp3*, *Brca1*, *Cdca8*, and *Thbs1* (Table 4 and Figure 6A).

**TABLE 4:** The hub-genes of DEmRNAs.

Gene symbol	LogFC	*p*-values	Gene symbol	LogFC	*P*-values
Polr2a	1.501	0.034	Ddb2	–2.264	0.027
Casp3	1.517	0.000	Pias1	–2.171	0.000
Brca1	1.601	0.009	Itgb3	–2.049	0.027
Cdca8	1.785	0.035	Ddx5	–1.843	0.017
Actb	1.945	0.011	Prkacb	–1.722	0.032
Itgam	2.784	0.021	Mapk1	–1.668	0.012
Thbs1	2.868	0.025	Itgb1	–1.593	0.010
/	/	/	Rbbp7	–1.519	0.016

DEmRNAs, differentially expressed mRNAs.

### Functional analysis of intersectional genes between differentially expressed long-non-coding RNAs *cis*-regulated target genes and differentially expressed mRNAs

**FIGURE 7:**
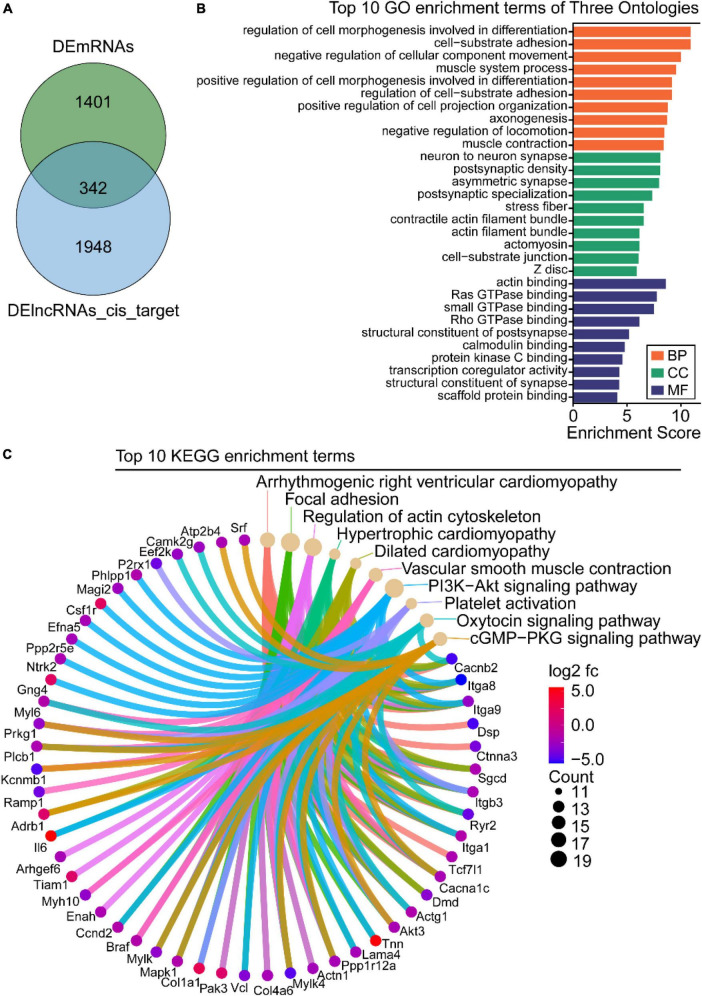
Gene ontology (GO) and Kyoto Encyclopedia of Genes and Genome (KEGG) enrichment analysis of the intersection between differentially expressed long-non-coding RNAs (DElncRNAs) *cis*-regulated target genes and differentially expressed mRNAs (DEmRNAs). **(A)** Venn diagram of DEmRNAs and DElncRNAs target genes. **(B)** GO analysis of the intersectional genes, the three ontologies mainly include CC, BP, and MF. **(C)** KEGG pathway analysis of the intersectional genes between DElncRNAs target genes and DEmRNAs.

Studies have reported that *cis*-regulation, *via* recruitment of remodeling factors to local chromatin, is one of the working modes of lncRNAs (21). To investigate the functions of DElncRNAs in regulating downstream genes in OSA-AD, genes located in 10 kbp downstream of DElncRNAs were identified as *cis*-regulatory genes and 2,290 genes were predicted. Furthermore, for the 1742 DEmRNAs and 2290 DElncRNAs, 342 *cis*-regulated genes were screened as intersectional genes using the Venn diagram (Figure 7A). Results from GO analysis showed that these genes were mostly enriched in vascular homeostasis-related terms in the BP, CC, and MF categories (Figure 7B), including “regulation of cell morphogenesis involved in differentiation,” “cell-substrate adhesion,” “negative regulation of cellular component movement,” “muscle system process,” “positive regulation of cell morphogenesis involved in differentiation,” “regulation of cell-substrate adhesion,” “-positive regulation of cell projection organization,” “negative regulation of locomotion,” “muscle contraction,” “stress fiber,” “actin filament bundle,” “contractile actin filament bundle,” “actin binding,” “scaffold protein binding,” and “GTPase binding.” KEGG analysis results showed that these genes were mainly enriched in cell apoptosis and vascular homeostasis-related terms (Figure 7C), such as “Focal adhesion,” “Regulation of actin cytoskeleton,” “Hypertrophic cardiomyopathy,” “Dilated cardiomyopathy,” “Vascular smooth muscle contraction,” “PI3K-Akt signaling pathway,” “Platelet activation,” “Oxytocin signaling pathway,” and “cGMP-PKG signaling pathway.”

**FIGURE 8:**
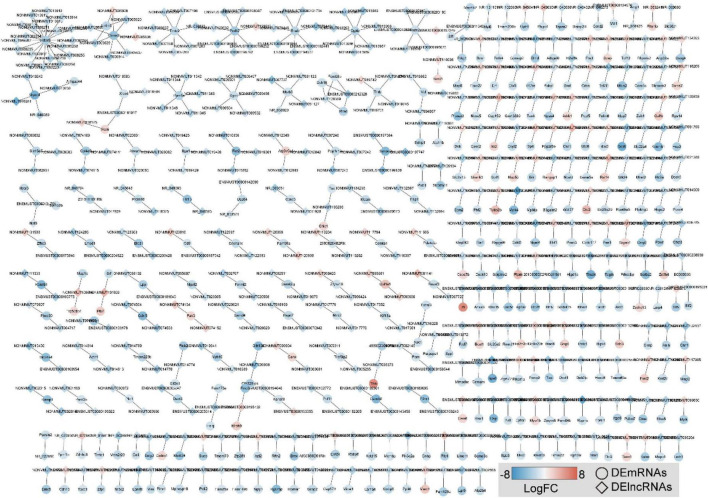
Construction of DE long-non-coding RNAs (DElncRNAs)-mediated *cis*-regulatory networks in aortic dissection (AD) tissues induced by chronic intermittent hypoxia (CIH) and Ang II. A total of 486 lncRNA–mRNA regulatory pairs were identified in AD tissues. The red to blue color gradient indicates the up- to downregulation of DE lncRNAs/mRNAs indicated by logFC; the circular nodes represent mRNAs, the rhombic nodes represent long-non-coding RNAs (lncRNAs).

To visually display a specific lncRNA *cis*-regulatory relationship in OSA-AD, we constructed DElncRNA mediated *cis*-regulatory networks using 342 genes and 486 lncRNA–mRNA regulatory pairs (Figure 8). The networks showed that *Hdac9* was *cis*-regulated by 19 DElncRNAs, including NON-MMUT013912, NON-MMUT013913, NON-MMUT013914, NON-MMUT013915, NON-MMUT013916, NON-MMUT013917, NON-MMUT088223, NON-MMUT088 230, NON-MMUT088234, NON-MMUT088235, NON-MMU T088244, NON-MMUT088252, NON-MMUT088254, NON-MMUT088255, NON-MMUT088258, NON-MMUT088265, NON-MMUT088267, NON-MMUT088268, and NON-MMUT 088271. In addition, *Lrrtm3* and *Ctnna3* were *cis*-regulated by the same DElncRNAs: NON-MMUT005920, NON-MMUT0 05925, NON-MMUT005926, and NON-MMUT005929.

### Functional analysis of differentially expressed circular RNAs host genes in obstructive sleep apnea-related aortic dissection

**FIGURE 9:**
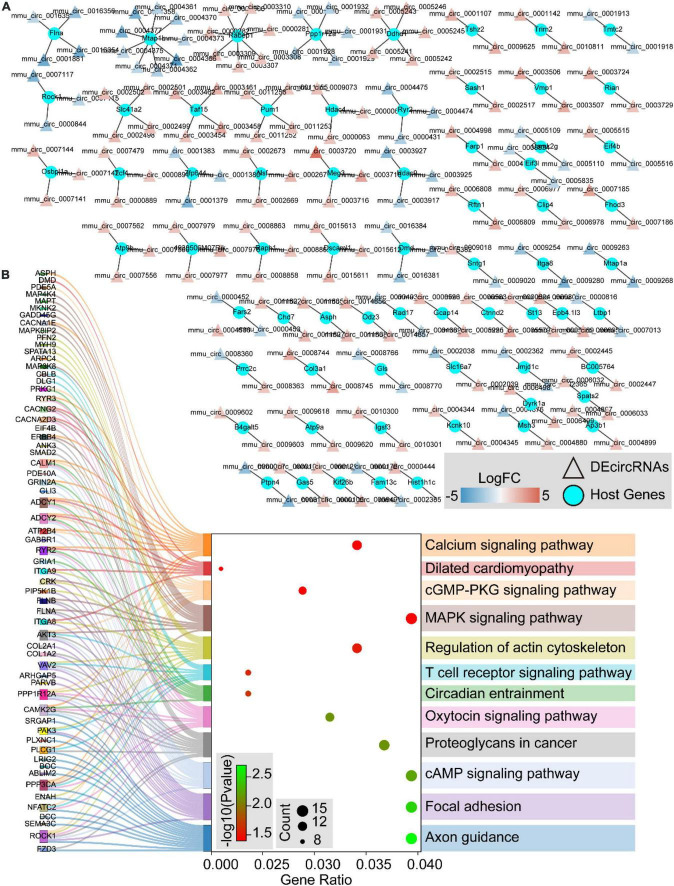
The host genes of differentially expressed circular RNAs (DEcircRNAs). **(A)** The networks of DEcircRNAs host genes in aortic dissection (AD) tissues induced by chronic intermittent hypoxia (CIH) and Ang II. The red to blue color gradient indicates up- to downregulation of DEcircRNAs, indicated by logFC; circular nodes represent mRNAs and triangular nodes represent circRNAs. **(B)** Sankey’s diagram showing the classification of DEcircRNAs host genes in AD tissues induced by CIH and Ang II, based on GO and KEGGs analyses.

We further traced the host genes of the 536 DEcircRNAs in OSA-AD, and obtained 421 host genes (Figure 9A). Notably, nine circRNAs (mmu_circ_0004377, mmu_circ_0004375, mmu_circ_0004373, mmu_circ_0004371, mmu_circ_0004370, mmu_circ_0004366, mmu_circ_0004362, mmu_circ_0004361, and mmu_circ_0004358) were transcribed from the host gene of *Mtap1b*. *Rabep1* was the common host gene for seven circRNAs, including mum_circ_0003310, mmu_circ_0003309, mmu_circ_0003308, mmu_circ_0003307, mmu_circ_0003306, mmu_circ_0000282, and mum_circ_0000281. Moreover, circRNAs from the same host gene had the same expression trend (Figure 9A). KEGG pathway enrichment analysis revealed that the 421 host genes were mainly involved in vasoactive, vessel structural homeostasis, and inflammatory terms (Figure 9B), including “Calcium signaling pathway,” “Focal adhesion,” “Regulation of actin cytoskeleton,” “cGMP-PKG signaling pathway,” “MAPK signaling pathway,” “T cell receptor signaling pathway,” and “cAMP signaling pathway.”

### Inflammation and apoptosis were widely found in human aortic dissection and the validation of hub genes in human aortic dissection

**FIGURE 10:**
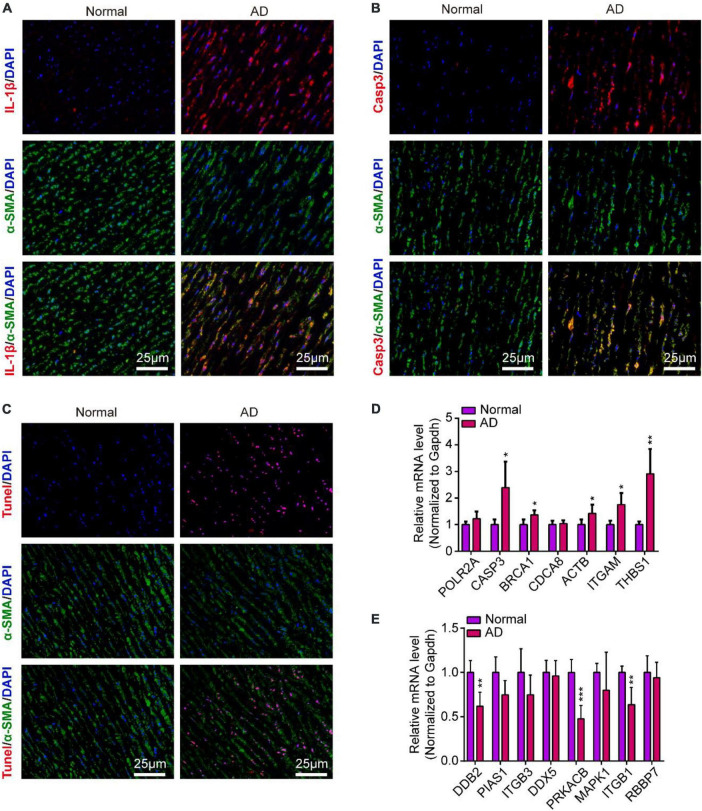
Inflammation and apoptosis were widely found in human aortic dissection and the validation of hub genes in human aortic dissection. **(A–C)** Representative images of immunofluorescence staining demonstrate Il-1β **(A)**, Casp3 **(B)** expression and Tunel positive cells **(C)** in human aortic tissues of aortic dissection (AD) and normal controls. Scale bar = 25 μm. **(D,E)** Quantitative real-time polymerase chain reaction (qRT-PCR) analysis show the expression of hub genes in human aortic tissues of AD and normal controls, including **(D)** upregulated hub genes and **(E)** downregulated hub genes. GAPDH was used as internal control; For normal group, *n* = 4, for AD group, *n* = 6; *P < 0.05, **P < 0.01, ***P < 0.001.

To validate the inflammation and apoptosis status in human aortic dissection and the conservation of hub genes, we used the human aortic dissected tissues to stained apoptotic and inflammatory indicators, including IL-1β, CASP3, and Tunel. As the data showed that the IL-1β was overexpressed in AD group compared to normal human aortic tissue (Figure 10A), indicated that the inflammatory response was accompanied in the process of AD. Meanwhile, the CASP3 protein and Tunel staining exhibited the cell apoptosis was activated in AD process (Figures 10B,C). What’s more, we detected the protein expressions of CASP3 and PARP1, which related to apoptosis and necrosis, in endothelial cells by Western blot analysis, and the data showed the apoptosis and necrosis were activated in hypoxia/Ang II treated endothelial cells (Supplementary Figure 1C). Then, we measured the expression of those hub genes in human AD tissues which were screened in CIH/Ang II-induced mouse AD. Our data suggested the expression of *CASP3*, *BRCA1*, *ACTB1*, *ITGAM*, *THBS1*, *DDB2*, *PRKACB*, and *ITGB1* in AD tissues shows species conservation (Figures 10D,E). This part suggested the inflammation and apoptosis were widely found in human aortic dissection and those hub genes show species conservation in human and mouse.

## Discussion

Aortic dissection is a cardiovascular emergency with high mortality and is defined by the progressive separation of the aortic layers by a hypertensive blood column, which leads to the degeneration of the aortic media (1). In the past two decades, the role of OSA in the development of AD has been taken seriously. Epidemiological investigations have reported that patients with AD present a higher OSA index (7–10). In patients with Marfan’s syndrome, the prevalence of OSA is much higher than that in matched controls. Moreover, aortic root diameters and mean aortic root diameters are significantly greater in patients with OSA (4.5 ± 0.6 cm) than in those without OSA (3.7 ± 0.6 cm) (12). These findings are consistent with those of Baguet et al. (11). Yanagi et al. (22) showed that patients with acute AD and OSA are characteristically tall, fat, and relatively young men with hypertension and indicated that OSA is a risk factor for acute AD in middle-aged men. Subsequent studies have consistently identified OSA as an important risk independent of AD, and oxygen therapy for the treatment of AD has been considered. A case report showed successful therapy in a patient with acute AD (De Bakey type 3b) combined with OSA using non-invasive positive pressure ventilation treatment (23). A molecular mechanism study disclosed that OSA facilitates the production of ROS, followed by HIF-α activation, induction of matrix metalloproteases expression, and eventually, progression of AD (14). Further evaluation of the molecular mechanism of OSA-AD is essential for the establishment of therapeutic strategies.

In this study, we first simulated OSA to establish the OSA-AD animal model using the CIH system. After 4 weeks of CIH and Ang II administration, 73.33% of mice had AD. This percentage was much higher than that for Ang II (26.67%) or CIH (12.50%) alone. Histological staining showed CIH and Ang II induced cystic medial degeneration in the aortic wall, which is characterized by the loss of elastic fibers and smooth muscle cells. Consistently, these features were reported by Humphrey et al. (24). To demonstrate the potential changes in transcript levels, we performed the SBC-ceRNA microarray assay, which is an effective method to study the molecular mechanism of disease (25–27). A total of 1,742 mRNAs, 2,625 lncRNAs, and 537 circRNAs were dysregulated in OSA-AD tissues, including 1,152 downregulated mRNAs, 590 upregulated mRNAs, 1,507 downregulated lncRNAs, 1,118 upregulated lncRNAs, 167 downregulated circRNAs, and 370 upregulated circRNAs. In addition, GO and KEGG enrichment analysis showed that DEmRNAs were mainly enriched in cell proliferation, cell migration, cell apoptosis, inflammatory response, aortic structure, and hypoxia-related terms.

Aortic vascular smooth muscle cells (SMCs) are regarded as the predominant cell type in the medial layer of the aortic wall and play a key role in maintaining structural stabilization (28). The proliferation and migration of SMCs occur in response to vascular injuries and contribute to the development of pathological remodeling and vascular diseases (29). Thus, SMC proliferation and maintenance of appropriate function have been thought to limit the progression of thoracic aortic aneurysm and dissection (30). In our previous study, we showed that Ang II had no significant effect on SMC proliferation (31); however, many studies have shown that hypoxia plays an important role in the proliferation of SMCs (32–34). Our data showed enrichment of “negative regulation of cell proliferation,” “positive regulation of smooth muscle cell proliferation,” “HIF-α signaling pathway,” and “response to hypoxia,” which suggests that the CIH-induced dysregulation of SMC proliferation leads to a loss of aortic middle cells in the OSA-AD model. In GO analysis, the term “positive regulation of cell migration” was enriched and studies have revealed that SMC migration was excessively activated during AD development. Jiang et al. (35) found that in SMCs, S100A12 increases the expression of migration-related MMP2/9 and VCAM-1 by activating the ERK1/2 signaling pathway in thoracic AD. Importantly, Liu et al. (14) showed that hypoxia facilitates the expression of matrix metalloproteases, mediated through HIF-α, and accelerates SMC migration during the development of AD. Therefore, targeting SMC migration signaling pathways may be a potential strategy for treating OSA-AD.

Growing evidence suggests that programmed cell death (apoptosis) plays a central role in SMC loss and AD (36, 37). Our GO and KEGG analyses revealed that 71 DEmRNAs were enriched for the term “apoptotic process” and 53 DEmRANs for “PI3K-Akt signaling pathway,” indicating that apoptosis plays a key role in OSA-AD. In a human study, the expression of the proapoptotic protein BAX was significantly elevated and that of the antiapoptotic protein BCL2L1 was remarkably decreased in AD tissues, indicating that the apoptotic index increased significantly in patients with AD (37). Yuan et al. (36) reported that BRG1 was upregulated in the SMCs of AD tissue, subsequently inducing SMC apoptosis and transition from contractile to synthetic phenotype. In human diseases, apoptosis is often accompanied by an inflammatory response. Not surprisingly, inflammatory terms were highlighted in our GO and KEGG enrichment analyses, including “Inflammatory response,” “-positive regulation of TNF production,” “neutrophil chemotaxis,” “Chemokine signaling pathway,” and “MAPK signaling pathway.” Using transgenic mice, Qiu et al. (38) disclosed that in SMCs, Sirt3 plays a protective role in AD by reducing ROS production, vascular inflammation, and apoptosis. Shen et al. (39) showed that the levels of AKT2 and phospho-AKT in human thoracic AD tissues were significantly reduced. Akt2-deficient mice displayed profound tissue destruction, which was accompanied by apoptotic cell death and inflammatory cell infiltration. Importantly, PPI analysis showed that most upregulated hub genes were related to inflammation and apoptosis. Casp3 is a key protein in the apoptotic pathway. Activated Casp3 degrades structural and functional proteins, which eventually induces cell death (40). Studies have shown that Casp3 is overexpressed in the aortic walls of patients with AD (41) and animal models (42, 43). Studies have found that Brca1 induced cell apoptosis mediated by ERK1/2 activation (44) and P53 signaling (45). Brca1 mediates the inflammatory response between no-tumor hepatitis/cirrhotic tissues and hepatocellular carcinoma (46). However, Singh et al. (47) found that BRCA1 expression was attenuated in the plaque region of human atherosclerotic carotid artery samples, and Brca1 deficiency led to greater inflammation-associated apoptosis and impaired endothelial function. In addition, many studies have reported that Cdca8 and Thbs1 are apoptosis and inflammation-related proteins (48–51). Thus, anti-apoptosis and anti-inflammation may be a potential therapeutic strategy for AD. Consistently, Ren et al. (52) alleviated AD by overexpressing IL-5 to reduce the inflammatory response and smooth muscle cell apoptosis.

Recent studies have indicated that the abnormal expression and functions of non-coding RNAs are closely related to AD (19). In this study, the SBC-ceRNA microarray assay demonstrated an abundance of DElncRNAs and DEcircRNAs in OSA-AD tissues, and included 1,507 downregulated lncRNAs, 1,118 upregulated lncRNAs, 167 downregulated circRNAs, and 370 upregulated circRNAs. KEGG pathway analysis showed that the *cis*-regulated genes of DElncRNAs and host genes of DEcircRNAs were mostly enriched in vessels with structural homeostasis-related terms. In other studies, most DElncRNAs and DEcircRNAs involved in the development of AD were enriched for cell proliferation, migration, apoptosis, and inflammation (17–19). For instance, the lncRNA H19 is involved in the development of AD by regulating SMC migration (18). LncRNA SENCR and lncRNA XIST were demonstrated to play roles in the development of AD by modulating SMC proliferation, migration, and apoptosis and maintaining the phenotypic switching of SMCs (53, 54). Although the functions of a few lncRNAs and circRNAs in AD are known, the functions of many DElncRNAs and DEcircRNAs have not been explained. Therefore, in the future, studies with non-coding RNAs can be used for developing potential therapeutic targets for OSA-AD.

## Conclusion

In summary, we established a *de novo* OSA-AD animal model using ApoE^–/–^ mice that were administered Ang II under CIH-hypoxia conditions. We performed an SBC-ceRNA microarray analysis to screen DEmRNAs, DElncRNAs, and DEcircRNAs for the first time in OSA-AD. GO and KEGG enrichment analysis demonstrated the key signaling pathways in OSA-AD. We discovered hub genes, *cis*-mediated genes, and host genes involved in the development of OSA-AD by constructing complex regulatory networks between the DEmRNAs, DElncRNAs, and DEcircRNAs, respectively. Therefore, our findings provide a basis for understanding the molecular mechanisms involved in the progression of OSA-AD and developing potential therapeutic strategies for OSA-AD.

## Data availability statement

The datasets analyzed for this study can be found in the GEO database, accession number: GSE215935.

## Ethics statement

The animal study was reviewed and approved by Tongji Medical College, Huazhong University of Science and Technology. Written informed consent was obtained from the individual(s) for the publication of any potentially identifiable images or data included in this article.

## Author contributions

JY, LH, and DS conceived and designed the study. YL, DS, and XM performed the experiments and analyzed the data. DS and YL wrote the manuscript. XM, SM, YJ, and DW modified the manuscript. All authors read the final manuscript and approved the submitted version.

## Footnotes

1
http://www.shbio.com/

2
http://www.ehbio.com/ImageGP

3
http://www.bioinformatics.com.cn

4
https://david.ncifcrf.gov

5
https://string-db.org/

## References

[1] Sayed A Munir M Bahbah EI. Aortic dissection: a review of the pathophysiology, management and prospective advances. *Curr Cardiol Rev.* (2021) 17:e230421186875. 10.2174/1573403X16666201014142930 33059568 PMC8762162

[2] Akutsu K. Etiology of aortic dissection. *Gen Thorac Cardiovasc Surg.* (2019) 67:271–6. 10.1007/s11748-019-01066-x 30689200

[3] Nienaber CA Clough RE Sakalihasan N Suzuki T Gibbs R Mussa F Aortic dissection. *Nat Rev Dis Primers.* (2016) 2:16053. 10.1038/nrdp.2016.53 27440162

[4] Yeghiazarians Y Jneid H Tietjens JR Redline S Brown DL El-Sherif N Obstructive sleep apnea and cardiovascular disease: a scientific statement from the American Heart Association. *Circulation.* (2021) 144:e56–67. 10.1161/CIR.0000000000000988 34148375

[5] Rana D Torrilus C Ahmad W Okam NA Fatima T Jahan N. Obstructive sleep apnea and cardiovascular morbidities: a review article. *Cureus.* (2020) 12:e10424. 10.7759/cureus.10424 32953361 PMC7494423

[6] Tietjens JR Claman D Kezirian EJ De Marco T Mirzayan A Sadroonri B Obstructive sleep apnea in cardiovascular disease: a review of the literature and proposed multidisciplinary clinical management strategy. *J Am Heart Assoc.* (2019) 8:e010440. 10.1161/JAHA.118.010440 30590966 PMC6405725

[7] Mo L He Q Wang Y Dong B He J. High prevalence of obstructive sleep apnea in Marfan’s syndrome. *Chin Med J.* (2014) 127:3150–5. 25189962

[8] Naito R Sakakura K Kasai T Dohi T Wada H Sugawara Y Aortic dissection is associated with intermittent hypoxia and re-oxygenation. *Heart Vessels.* (2012) 27:265–70. 10.1007/s00380-011-0149-x 21573950

[9] Saruhara H Takata Y Usui Y Shiina K Hashimura Y Kato K Obstructive sleep apnea as a potential risk factor for aortic disease. *Heart Vessels.* (2012) 27:166–73. 10.1007/s00380-011-0135-3 21442254

[10] Sampol G Romero O Salas A Tovar JL Lloberes P Sagales T Obstructive sleep apnea and thoracic aorta dissection. *Am J Respir Crit Care Med.* (2003) 168:1528–31. 10.1164/rccm.200304-566OC 12904327

[11] Baguet JP Minville C Tamisier R Roche F Barone-Rochette G Ormezzano O Increased aortic root size is associated with nocturnal hypoxia and diastolic blood pressure in obstructive sleep apnea. *Sleep.* (2011) 34:1605–7. 10.5665/sleep.1406 22043131 PMC3198215

[12] Kohler M Blair E Risby P Nickol AH Wordsworth P Forfar C The prevalence of obstructive sleep apnoea and its association with aortic dilatation in Marfan’s syndrome. *Thorax.* (2009) 64:162–6. 10.1136/thx.2008.102756 18852161

[13] Gaisl T Rejmer P Roeder M Baumgartner P Sievi NA Siegfried S Obstructive sleep apnoea and the progression of thoracic aortic aneurysm: a prospective cohort study. *Eur Respir J.* (2021) 57:2003322. 10.1183/13993003.03322-2020 33214207

[14] Liu W Zhang W Wang T Wu J Zhong X Gao K Obstructive sleep apnea syndrome promotes the progression of aortic dissection via a ROS- HIF-1alpha-MMPs associated pathway. *Int J Biol Sci.* (2019) 15:2774–82. 10.7150/ijbs.34888 31853217 PMC6909961

[15] Altesha MA Ni T Khan A Liu K Zheng X. Circular RNA in cardiovascular disease. *J Cell Physiol.* (2019) 234:5588–600. 10.1002/jcp.27384 30341894

[16] Uchida S Dimmeler S. Long noncoding RNAs in cardiovascular diseases. *Circ Res.* (2015) 116:737–50. 10.1161/CIRCRESAHA.116.302521 25677520

[17] Zhang S Zhao S Han X Zhang Y Jin X Yuan Y Lnc-C2orf63-4-1 Confers VSMC homeostasis and prevents aortic dissection formation via STAT3 interaction. *Front Cell Dev Biol.* (2021) 9:792051. 10.3389/fcell.2021.792051 34938738 PMC8685433

[18] Ren M Wang T Wei X Wang Y Ouyang C Xie Y LncRNA H19 regulates smooth muscle cell functions and participates in the development of aortic dissection through sponging miR-193b-3p. *Biosci Rep.* (2021) 41:BSR20202298. 10.1042/BSR20202298 33403385 PMC7823186

[19] Cheng M Yang Y Xin H Li M Zong T He X Non-coding RNAs in aortic dissection: from biomarkers to therapeutic targets. *J Cell Mol Med.* (2020) 24:11622–37. 10.1111/jcmm.15802 32885591 PMC7578866

[20] Sun D Xiang G Wang J Li Y Mei S Ding H miRNA 146b-5p protects against atherosclerosis by inhibiting vascular smooth muscle cell proliferation and migration. *Epigenomics.* (2020) 12:2189–204. 10.2217/epi-2020-0155 33084403

[21] Guil S Esteller M. Cis-acting noncoding RNAs: friends and foes. *Nat Struct Mol Biol.* (2012) 19:1068–75. 10.1038/nsmb.2428 23132386

[22] Yanagi H Imoto K Suzuki S Uchida K Masuda M Miyashita A. Acute aortic dissection associated with sleep apnea syndrome. *Ann Thorac Cardiovasc Surg.* (2013) 19:456–60. 10.5761/atcs.oa.12.02014 23328110

[23] Yamashita S Dohi T Narui K Momomura S. Therapeutic efficacy of continuous positive airway pressure in obstructive sleep apnea patients with acute aortic dissection: a case report. *J Atheroscler Thromb.* (2010) 17:999–1002. 10.5551/jat.4895 20610893

[24] Humphrey JD Schwartz MA Tellides G Milewicz DM. Role of mechanotransduction in vascular biology: focus on thoracic aortic aneurysms and dissections. *Circ Res.* (2015) 116:1448–61. 10.1161/CIRCRESAHA.114.304936 25858068 PMC4420625

[25] Tang X Deng Z Ding P Qiang W Lu Y Gao S A novel protein encoded by circHNRNPU promotes multiple myeloma progression by regulating the bone marrow microenvironment and alternative splicing. *J Exp Clin Cancer Res.* (2022) 41:85. 10.1186/s13046-022-02276-7 35260179 PMC8903708

[26] Zhang Y Han T Li J Cai H Xu J Chen L Comprehensive analysis of the regulatory network of differentially expressed mRNAs, lncRNAs and circRNAs in gastric cancer. *Biomed Pharmacother.* (2020) 122:109686. 10.1016/j.biopha.2019.109686 31786464

[27] Jia N Tong H Zhang Y Katayama H Wang Y Lu W CeRNA expression profiling identifies KIT-related circRNA-miRNA-mRNA networks in gastrointestinal stromal tumour. *Front Genet.* (2019) 10:825. 10.3389/fgene.2019.00825 31552107 PMC6746987

[28] Korneva A Humphrey JD. Maladaptive aortic remodeling in hypertension associates with dysfunctional smooth muscle contractility. *Am J Physiol Heart Circ Physiol.* (2019) 316:H265–78. 10.1152/ajpheart.00503.2017 30412437 PMC6397387

[29] Wang D Wang Q Yan G Qiao Y Tang C. Phloretin inhibits platelet-derived growth factor-BB-induced rat aortic smooth muscle cell proliferation, migration, and neointimal formation after carotid injury. *J Cardiovasc Pharmacol.* (2015) 65:444–55. 10.1097/FJC.0000000000000213 25945863

[30] Li G Wang M Caulk AW Cilfone NA Gujja S Qin L Chronic mTOR activation induces a degradative smooth muscle cell phenotype. *J Clin Invest.* (2020) 130:1233–51. 10.1172/JCI131048 32039915 PMC7269581

[31] Sun D Zhang M Li Y Mei S Qin J Yan J. c-Jun/Ap-1 is upregulated in an Ang IIinduced abdominal aortic aneurysm formation model and mediates Chop expression in mouse aortic smooth muscle cells. *Mol Med Rep.* (2019) 19:3459–68. 10.3892/mmr.2019.10017 30864718 PMC6472129

[32] Seong M Kang H. Hypoxia-induced miR-1260b regulates vascular smooth muscle cell proliferation by targeting GDF11. *BMB Rep.* (2020) 53:206–11. 10.5483/BMBRep.2020.53.4.136 31818357 PMC7196185

[33] Zhao Q Sun D Li Y Qin J Yan J. Integrated analyses of lncRNAs microarray profiles and mRNA-lncRNA coexpression in smooth muscle cells under hypoxic and normoxic conditions. *Biosci Rep.* (2019) 39:BSR20181783. 10.1042/BSR20181783 30850398 PMC6443952

[34] Schultz K Fanburg BL Beasley D. Hypoxia and hypoxia-inducible factor-1alpha promote growth factor-induced proliferation of human vascular smooth muscle cells. *Am J Physiol Heart Circ Physiol.* (2006) 290:H2528–34. 10.1152/ajpheart.01077.2005 16399861

[35] Jiang W Wang Z Hu Z Wu H Hu R Hu X Blocking the ERK1/2 signal pathway can inhibit S100A12 induced human aortic smooth muscle cells damage. *Cell Biol Int.* (2017) 41:1307–15. 10.1002/cbin.10840 28816402

[36] Yuan Y Wang C Xu J Tao J Xu Z Huang S. BRG1 overexpression in smooth muscle cells promotes the development of thoracic aortic dissection. *BMC Cardiovasc Disord.* (2014) 14:144. 10.1186/1471-2261-14-144 25304030 PMC4531522

[37] Durdu S Deniz GC Balci D Zaim C Dogan A Can A Apoptotic vascular smooth muscle cell depletion via BCL2 family of proteins in human ascending aortic aneurysm and dissection. *Cardiovasc Ther.* (2012) 30:308–16. 10.1111/1755-5922.12007 22978789

[38] Qiu L Yi S Yu T Hao Y. Sirt3 protects against thoracic aortic dissection formation by reducing reactive oxygen species, vascular inflammation, and apoptosis of smooth muscle cells. *Front Cardiovasc Med.* (2021) 8:675647. 10.3389/fcvm.2021.675647 34095262 PMC8176563

[39] Shen YH Zhang L Ren P Nguyen MT Zou S Wu D AKT2 confers protection against aortic aneurysms and dissections. *Circ Res.* (2013) 112:618–32. 10.1161/CIRCRESAHA.112.300735 23250987 PMC3586338

[40] Jiang M Qi L Li L Li Y. The caspase-3/GSDME signal pathway as a switch between apoptosis and pyroptosis in cancer. *Cell Death Discov.* (2020) 6:112. 10.1038/s41420-020-00349-0 33133646 PMC7595122

[41] Lee JH Kim J Lee SJ Kim YA Maeng YI Park KK. Apoptosis and fibrosis of vascular smooth muscle cells in aortic dissection: an immunohistochemical study. *Int J Clin Exp Pathol.* (2020) 13:1962–9. 32922591 PMC7476953

[42] Shi Y Liu B Wang CS Yang CS. MST1 down-regulation in decreasing apoptosis of aortic dissection smooth muscle cell apoptosis. *Eur Rev Med Pharmacol Sci.* (2018) 22:2044–51. 10.26355/eurrev_201804_14734 29687861

[43] Das D Gawdzik J Dellefave-Castillo L McNally EM Husain A Raman J S100A12 expression in thoracic aortic aneurysm is associated with increased risk of dissection and perioperative complications. *J Am Coll Cardiol.* (2012) 60:775–85. 10.1016/j.jacc.2012.04.027 22818064 PMC3422448

[44] Yan Y Haas JP Kim M Sgagias MK Cowan KH. BRCA1-induced apoptosis involves inactivation of ERK1/2 activities. *J Biol Chem.* (2002) 277:33422–30. 10.1074/jbc.M201147200 12082091

[45] Zhang W Luo J Chen F Yang F Song W Zhu A BRCA1 regulates PIG3-mediated apoptosis in a p53-dependent manner. *Oncotarget.* (2015) 6:7608–18. 10.18632/oncotarget.3263 25797244 PMC4480703

[46] Diao H Wang L Huang J Jiang M Zhou H Li X BRCA1-mediated inflammation and growth activated & inhibited transition mechanisms between no-tumor hepatitis/cirrhotic tissues and HCC. *J Cell Biochem.* (2014) 115:641–50. 10.1002/jcb.24699 24151232

[47] Singh KK Shukla PC Quan A Al-Omran M Lovren F Pan Y BRCA1 is a novel target to improve endothelial dysfunction and retard atherosclerosis. *J Thorac Cardiovasc Surg.* (2013) 146:949–60.e4. 10.1016/j.jtcvs.2012.12.064 23415688

[48] Yao Y Zhang X Xu J Gao F Wu Y Cui X circ_014260/miR-384/THBS1 aggravates spinal cord injury in rats by promoting neuronal apoptosis and endoplasmic reticulum stress. *Am J Transl Res.* (2022) 14:518–33. 35173872 PMC8829636

[49] Li Q Liang J Chen B. Identification of CDCA8, DSN1 and BIRC5 in regulating cell cycle and apoptosis in osteosarcoma using bioinformatics and cell biology. *Technol Cancer Res Treat.* (2020) 19:1533033820965605. 10.1177/1533033820965605 33153400 PMC7673055

[50] Gao X Wen X He H Zheng L Yang Y Yang J Knockdown of CDCA8 inhibits the proliferation and enhances the apoptosis of bladder cancer cells. *PeerJ.* (2020) 8:e9078. 10.7717/peerj.9078 32377458 PMC7194097

[51] Zhu W Yang M Shang J Xu Y Wang Y Tao Q MiR-222 inhibits apoptosis in porcine follicular granulosa cells by targeting the THBS1 gene. *Anim Sci J.* (2019) 90:719–27. 10.1111/asj.13208 30983045

[52] Ren W Wang Z Wang J Wu Z Ren Q Yu A IL-5 overexpression attenuates aortic dissection by reducing inflammation and smooth muscle cell apoptosis. *Life Sci.* (2020) 241:117144. 10.1016/j.lfs.2019.117144 31830482

[53] Zhang X Wu H Mai C Qi Y. Long noncoding RNA XIST/miR-17/PTEN axis modulates the proliferation and apoptosis of vascular smooth muscle cells to affect stanford type a aortic dissection. *J Cardiovasc Pharmacol.* (2020) 76:53–62. 10.1097/FJC.0000000000000835 32282501

[54] Song Y Wang T Mu C Gui W Deng Y Ma R. LncRNA SENCR overexpression attenuated the proliferation, migration and phenotypic switching of vascular smooth muscle cells in aortic dissection via the miR-206/myocardin axis. *Nutr Metab Cardiovasc Dis.* (2022) 32:1560–70. 10.1016/j.numecd.2022.03.004 35351345

