# Supplementary material for: mRNA, lncRNA, and circRNA expression profiles in a new aortic dissection murine model induced by hypoxia and Ang II
Source: Front Cardiovasc Med. 2022 Oct 26;9:984087. doi: 10.3389/fcvm.2022.984087 (PMC9643159; doi:10.3389/fcvm.2022.984087)
Supplement: Supplementary file 1 [file Data_Sheet_1.PDF]

Supplementary Data

mRNA, lncRNA, and CircRNA expression profiles in a new aortic dissection murine model induced by hypoxia and Ang II

Yuanyuan Li[1,2,#], Xiaozhu Ma[1,#], Shuai Mei[1], Yueping Ji[3], Dong Wang[3], Liqun He[3,*], Dating Sun[1,3,*], Jiangtao Yan[1,*]

[1]Department of Cardiology, Tongji Hospital, Tongji Medical College, Huazhong University of Science and Technology, Wuhan, 430030, People's Rep. of China

[2]Department of Rheumatology and Immunology, Tongji Hospital, Tongji Medical College, Huazhong University of Science and Technology, Wuhan, 430030, People's Rep. of China

[3]Department of Cardiology, Wuhan No. 1 Hospital, Wuhan Hospital of Traditional Chinese and Western Medicine, Wuhan, 430022, People's Rep. of China

[#]These authors contribute equally to this work

***Correspondence:**

Jiangtao Yan: jtyan_tjh@163.com

Dating Sun: sundating0407@163.com

Liqun He: Liqunhe0902@163.com

Supplementary Figure

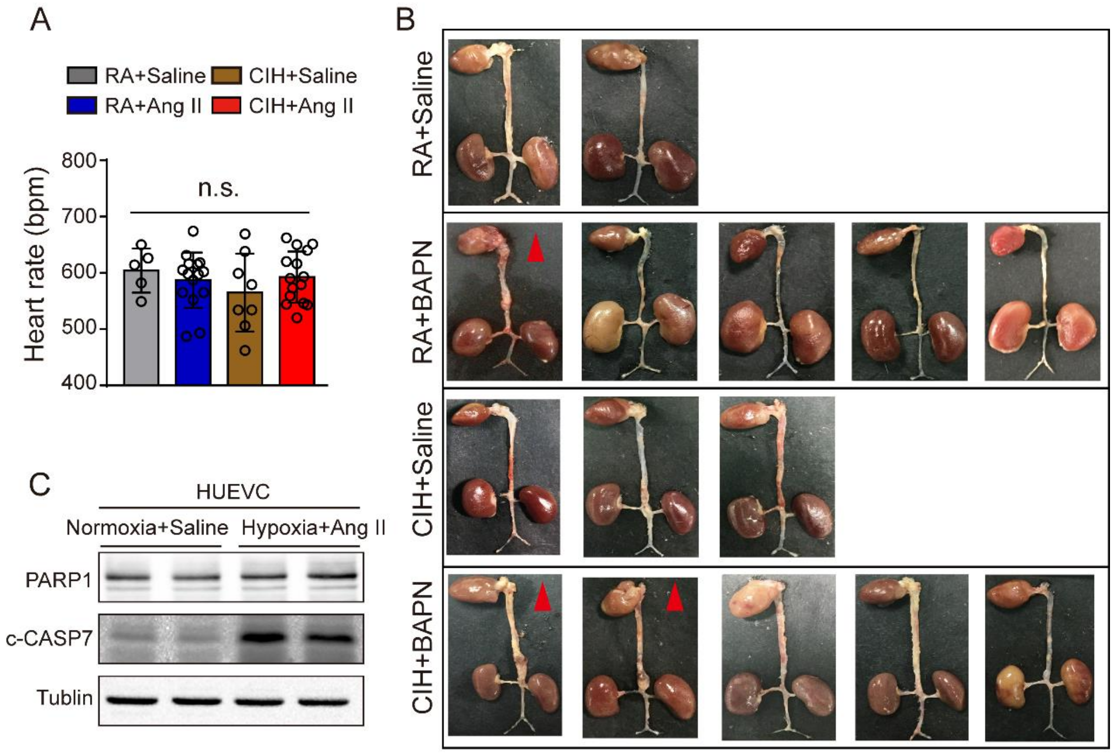

Furgure S1. (A) mice heart rate in four group. n = 5–15 mice per group; n.s., not significant; (B) Whole aorta tissue from the indicated mice after CIH/RA and BAPN/saline administration. The red triangle shows mice with aortic accident. (F) Western blot showing the apoptosis and necrosis related protein expression in HUEVC cells induced by hypoxia and Ang II. Tublin was used as control.

Supplementary Tables

Table S1. Primers for qRT-PCR (mouse)

Genesymbol	Forward sequence: 5' to 3'	Reverse sequence: 5' to 3'
Polr2a	AAATACCCAGAAACAACGGAGG	CCAGTCCGCTCAATCACCC
Casp3	TGAAGGGGTCATTTATGGGACA	CCAGTCAGACTCCGGCAGTA
Brca1	CGAATCTGAGTCCCCTAAAGAGC	AAGCAACTTGACCTTGGGGTA
Cdca8	AGCATCTGTGTGCCTCACTG	ATGAACTGCCATGTTTGCCGA
Actb	GGCTGTATTCCCCTCCATCG	CCAGTTGGTAACAATGCCATGT
Itgam	ATGGACGCTGATGGCAATACC	TCCCCATTCACGTCTCCCA
Thbs1	GGGGAGATAACGGTGTGTTTG	CGGGGATCAGGTTGGCATT
Ddb2	TCCGAATGCACAAGAAGAAAGT	CACTGTTTGATCTATGGAGGCTG
Pias1	ACGCAAACACGAACTTCTTACA	TCCGCAGGCGTCATAATTTC
Itgb3	CCCCGATGTAACCTGAAGGAG	GAAGGGCAATCCTCTGAGGG
Ddx5	TTCCTGCGAATGTCATGGATG	TCCAGTCTGAGCTACTCCAAC
Prkacb	ATGGTTATGGAATACGTCCCTGG	AATTAAGAGGTTTTCCGGCTTGA
Mapk1	GCCTTACTCTACTTCTCCCCA	CTGCCTCTGACTTCTGAATGC
Itgb1	ACTGTGATGCCGTATATTAGCAC	GATATGCGTTGCTGACCAACA
Rbbp7	GAGCGTGTCATCAACGAAGAG	CCACTGAACGGTAAGACTGGG
Gapdh	AGGTCGGTGTGAACGGATTTG	TGTAGACCATGTAGTTGAGGTCA

Table S2. Primers for qRT-PCR (human)

Genesymbol	Forward sequence: 5' to 3'	Reverse sequence: 5' to 3'
POLR2A	GGGTGGCATCAAATACCCAGA	AGACACAGCGCAAAACTTTCA
CASP3	CATGGAAGCGAATCAATGGACT	CTGTACCAGACCGAGATGTCA
BRCA1	GAAACCGTGCCAAAAGACTTC	CCAAGGTTAGAGAGTTGGACAC
CDCA8	GAAGGGCAGTAGTCGGGTG	TCACGGTCGAAGTCTTTCAGA
ACTB	CATGTACGTTGCTATCCAGGC	CTCCTTAATGTCACGCACGAT
ITGAM	GCCTTGACCTTATGTCATGGG	CCTGTGCTGTAGTCGCACT
THBS1	GCCATCCGCACTAACTACATT	TCCGTTGTGATAGCATAGGGG
DDB2	ACCTCCGAGATTGTATTACGCC	TCACATCTTCTGCTAGGACCG
PIAS1	ACAGTGCGGAACTAAAGCAAA	GGACTTGAATGTACGTTGGGG
ITGB3	CATGAAGGATGATCTGTGGAGC	AATCCGCAGGTTACTGGTGAG
DDX5	ATGTCGGGTTATTCGAGTGACC	TGTGCGCCTAGCCAAATCAG
PRKACB	CCATGCACGGTTCTATGCAG	GTCTGTGACCTGGATATAGCCTT
MAPK1	TACACCAACCTCTCGTACATCG	CATGTCTGAAGCGCAGTAAGATT
ITGB1	CCTACTTCTGCACGATGTGATG	CCTTTGCTACGGTTGGTTACATT
RBBP7	GAGGAGCGTGTCATCAATGAA	GCATGGGTCATAACCAGGTCATA
GAPDH	GGAGCGAGATCCCTCCAAAAT	GGCTGTTGTCATACTTCTCATGG
